# miR-19-3p Promotes Autophagy and Apoptosis in Pelvic Organ Prolapse Through the AKT/mTOR/p70S6K Pathway: Function of miR-19-3p on Vaginal Fibroblasts by Targeting IGF-1

**DOI:** 10.1097/SPV.0000000000001034

**Published:** 2021-02-11

**Authors:** Yitong Yin, Meiying Qin, Meng Luan, Zhijun Xia

**Affiliations:** From the Department of Obstetrics and Gynecology, Pelvic Floor Disease Diagnosis and Treatment Center, Shengjing Hospital of China Medical University, Shenyang, China.

**Keywords:** miR-19-3p, IGF-1, pelvic organ prolapse, fibroblast

## Abstract

**Objective:**

Pelvic organ prolapse (POP) is a common condition in older women. A decrease in collagen 1 (Col-1) expression is one of the main causes of POP. Many microRNAs play an important role in regulating target genes. The relationship between miR-19-3p and POP is investigated in this study, and the molecular mechanism was also explored to find whether miR-19-3p may be a potential target for early diagnosis and prevention of POP.

**Methods:**

A total of 60 patients with POP and 60 patients without POP were included in this study. Reverse transcription-polymerase chain reaction and Western blot were used to detect the expression of miR-19-3p, insulin-like growth factor 1 (IGF-1), and the Akt/mTOR/p70S6K pathway. Cell cycle was defined by flow cytometric analysis. The combination of miR-19-3p and IGF-1 was revealed by luciferase assays.

**Results:**

The results of this study show that miR-19-3p was upregulated in the tissue of patients with POP, whereas COL-1 and IGF-1 expressions were lower in the POP group. miR-19-3p promoted excessive fibroblast autophagy and apoptosis. miR-19-3p negatively regulated the Akt/mTOR/p70S6K pathway and inhibited COL-1 secretion. Luciferase reporter assay showed that miR-19-3p regulated IGF-1 expression by direct target binding.

**Conclusions:**

miR-19-3p has negative associations with the expression of Col-1. Our study highlights that miR-19-3p may affect the synthesis of Col-1 by targeting IGF-1 and that it may play an vital role in POP.

Pelvic organ prolapse (POP) is a common condition in middle-aged and older women. A study by Islam et al^1^ showed that the incidence of POP is 8%–36%. The relaxation of pelvic floor support, such as pelvic floor ligaments and fascia, is considered to be the main cause of POP. Many studies have suggested that a decrease in pelvic floor collagen tissue, especially type I collagen, is the important factor causing relaxation of the pelvic floor supporting structure.^2^ Fibroblasts are the main functional cells that secrete collagen. The number and function of fibroblasts in pelvic floor tissue are positively correlated with collagen secretion.^3^ Ligament relaxation caused by reduced collagen secretion can be prevented and treated by early diagnosis and early intervention. Therefore, furthering understanding of the molecular mechanism of the disease is potentially of great significance for preventing and treating this condition.

Autophagy is a conserved cellular activity in the evolution of eukaryotic cells. Moderate autophagy can maintain cell homeostasis, but excessive persistent autophagy may lead to type II programmed cell death.^4^ Mammalian target of rapamycin (mTOR) is a serine/threonine protein kinase closely related to autophagy. mTOR is affected by many factors, including growth factor, energy status, internal environment, and stress.^5^ When the internal environment is stable, mTOR is activated by many cascades, including the PI3K-AKT pathway, which inhibits autophagy. Conversely, mTOR may be inhibited when the level of growth factor decreases, and autophagy may be initiated by downstream p70S6K protein. So far, there has been no report on the correlation between AKT/mTOR/p70S6K-related autophagy and POP.

Insulin-like growth factor 1 (IGF-1) is a mitogen and apoptotic inhibitor^6^ that participates in a large number of physiological processes, including the promotion of bone and cartilage tissue healing,^7^ the promotion of muscle tissue growth,^8^ and the promotion of tendon tissue and skin tissue proliferation and healing.^9^ IGF-1 can also stimulate the fibroblasts to secrete extracellular matrix molecules, such as polysaccharide protein and collagen I (Col-1).^10^ IGF-1 promotes the self-phosphorylation of IGF-1 receptors by binding to the IGF-1 receptor on the surface of the cell membrane, after which it activates the insulin receptor substrate-1 and the downstream PI3K/AKT pathway.^11^

MicroRNA (miRNA) is a noncoding small RNA, which is usually approximately 20 nucleotides in length. Many miRNAs recognize the 5′-untranslated region (UTR) of the target gene by incomplete complementary pairing, which affects the translation of target genes. Some miRNAs are involved in regulating cellular activities, such as fibrosis or inflammation.^12^ miR-19-3p was found to be related to tissue fibrosis.^13^ Downregulation of miR-19-3p was found to lead to intestinal fibrosis and arthrofibrosis.^14^ We speculated that miR-19-3p might be a regulator of fibroblast function, and IGF-1 may be its target gene.

The objectives of this study were to describe the relationship between miR-19-3p and the POP condition, to explore the molecular mechanism(s) between them, and to find a potential means of early diagnosis and prevention of POP.

## STUDY DESIGN

### Study Samples

Vaginal wall biopsy specimens were obtained from 60 patients with POP III–IV who underwent pelvic reconstruction surgery and 60 non-POP patients who underwent hysterectomies because of benign gynecological diseases in Shengjing Hospital of China Medical University. The pelvic organ prolapse quatitative evaluation system was used for staging.^15^ Pelvic examinations were performed by the same examiner. The exclusion criteria were collagen metabolism disease, hormone therapy history (including estrogen therapy vaginally), and vaginal surgery history.

This study was approved by the Ethics Committee of the China Medical University–affiliated Sheng Jing Hospital, Shenyang, China. All patients gave written informed consent for the use of the samples in this study.

Approximately 1.0 × 1.0-cm-size full-thickness samples were obtained from the fornix of the vagina (at POP-Q point D) during surgery to standardize the vaginal biopsy site. The vaginal samples were washed in ice-cold phosphate-buffered saline solution, and they were immediately frozen in liquid nitrogen and stored at −80°C.

### Primary Fibroblast Culture and Identification

The vaginal tissue was cut into 1 × 1 × 1-mm pieces, the epithelium of the vaginal mucosa was removed, and it was then digested in trypsase for 120 minutes, put through a centrifuged at 1,000 rpm for 8 minutes, and finally removed from the trypsase. Then the cells were plated and cultured in Dulbecco modified Eagle medium, with 20% fetal bovine serum, 100 μg/mL streptomycin, and 100 IU/mL penicillin in 5% CO_2_ at 37°C. Cells ranging from passages 4 to 6 were used in the experiments. Human vaginal fibroblasts were identified by antivimentin antibody staining.

### miRNA Mimic and Plasmid Transfection

An miR-19-3p mimic, an miR-19-3p inhibitor, and a negative control (NC) were purchased from RiboBio (Guangzhou, China). Cells were plated at 50% confluence and transfected with 100 nM miR-19-3p mimic, miR-19-3p inhibitor, or NC using Lipofectamine 3000 (Invitrogen, Carlsbad, California) according to the manufacturer’s instructions.

### Western Blot Analysis

Cells were harvested and dissolved in a RIPA lysis buffer containing protease and phosphatase inhibitor. The cell protein lysates were centrifuged at 12,000 rpm for 10 minutes. Furthermore, 30 μg proteins were separated by a 10% sodium dodecyl sulfate–polyacrylamide gel and electrotransferred to a polyvinylidene difluoride membrane. The proteins were incubated overnight at 4°C with specific antibodies, including AKT, mTOR, p70S6K, p-AKT, p-mTor, and p-p70S6K. Then the proteins were further incubated with a corresponding horseradish peroxidase–linked secondary antibody. Gray levels were detected on the enhanced chemiluminescence imaging system. For the tissue sample, the samples were homogenized in a 200 μL RIPA buffer with protease inhibitor; the subsequent step process was almost exactly the same as the Western blot for cells. The proteins were detected with specific antibodies including Col-1 and IGF-1.

### Real-Time Quantitative Reverse Transcription-Polymerase Chain Reaction

Total RNA was prepared from cells or the crushed tissue using TRIzol reagent (Life Technologies, Carlsbad, California). Furthermore, 1 μg of RNA was used as a template to synthesize cDNA using the reverse transcription kit (Takara, Shiga, Japan). Quantitative reverse transcription-polymerase chain reaction (qRT-PCR) was performed according to the directions of SYBR Green qPCR KIT (Takara). The cycling temperatures and times were as follows: initial denaturation at 95°C for 10 minutes, followed by 40 cycles at 95°C for 30 seconds, 56°C for 30 seconds, and 72°C for 30 seconds. The relative expression of genes was normalized to GAPDH or U6. The primer sequences are shown in Table 1.

**TABLE 1:** Primer Sequences

Targeted Gene	Direction and Sequence
*miR-19-3p*	F: 5′-AATAACTAGTAGGAAGCACTGTTGGAGCTACTG-3′
	R: 5′-ATAAGCGGCCGCAGTCACCAAAATGTATTATAA-3′
*IGF-1*	F: 5′-ACGCTGTCTACCAGATTCCC-3′
	R: 5′-TGCCCACAAACTCCTCAAAC-3′
*COL-1*	F: 5′-CCTGGATGCCATCAAAGTCT-3′
	R: 5′-AATCCATCGGTCATGCTCTC-3′
*U6*	F: 5′⁃CTCGCTTCG⁃GCAGCACA⁃3′
	R: 5′⁃AACGCTTCACGA⁃ATTTGCGT⁃3
*GAPDH*	F: 5′-CCAGGTGGTCTCCTCTGA-3′
	R: 5′-GCTGTAGCCAAATCGTTGT-3′

### Flow Cytometric Analysis

Cell apoptosis was assayed using the Annexin V–EGFP/PI Apoptosis Detection Kit (KeyGEN BioTECH, China). Fibroblasts were treated with different concentrations of miR-19-3p (0, 50, and 100 nM), and then the fibroblasts were harvested and resuspended in binding a buffer at a concentration of 1 × 10^6^ cells/mL. Cells were stained with Annexin V–EGFP (5 μL) and propidium iodide (5 μL). Then, the proportion of cells in different stages was determined using a flow cytometer (Beyotime, China).

### Fluorescence in Situ Hybridization Assay

The probe of miR-19-3p was designed to determine different expressions of vaginal tissue in POP and non-POP patients. Following the previous experimental instruction, the tissue was embedded and sliced, and then baked at 62°C in an oven for 2 hours. Xylene dewaxing was performed, followed by soaking in 100%, 85%, and 70% ethanol for 3 minutes, and distilled water for 5 minutes. The repair solution was boiled for 10 minutes, digested by Proteinase K for 10 minutes at 37°C, and then incubated with 6 ng/μL miR-19-3p probe at 37°C overnight. The primary antibody was added to the slide at 1:200 dilution. Cell nuclei were stained with DAPI (4′,6-diamidino-2-phenylindole) after incubation of the secondary antibody. Images were acquired using a fluorescence microscope.

### Enzyme-Linked Immunosorbent Assay

The level of COL-1 secreted by the fibroblasts was measured by enzyme-linked immunosorbent assay (ELISA) kits (Yi Fei Xue Biotechnology, Nanjing, China) according to a standard procedure.

### Luciferase Reporter Assay

IGF-1 oligonucleotides, in which the miR-19-3p binding sites were present or deleted (wild type or mutant type), were amplified and cloned using a pGL3 basic luciferase reporter vector (Promega, Madison, Wisconsin). Then the IGF-1–3′-UTR inserted luciferase reporter vectors or control vectors were cotransfected with the miR-19-3p mimic or NC using Lipofectamine 2000. After 48 hours, luciferase activity was assayed using the Dual-Glo Luciferase Assay System (Promega).

### Statistical Analysis

SPSS19.0 was used for data analysis (SPSS, Chicago, Illinois). All data have been presented as means±SD. Student *t* test and 1-way analysis of variance were performed for paired comparisons and multiple comparisons, respectively. *P* < 0.05 was considered statistically significant.

## RESULTS

### COL-1 and IGF-1 Expressions Were Lower in the POP Group. miR-19-3p Expression Was Increased in the Tissue of Patients With POP

**FIGURE 1:**
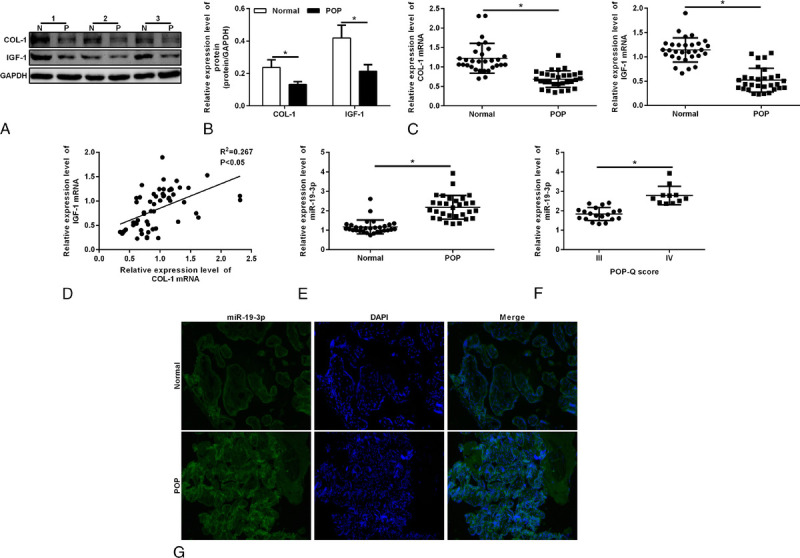
A, Protein expressions of COL-1 and IGF-1 in the POP group and control group. Protein was qualified by means of Western blotting. P stands for the POP group; and N, for the normal group. B, Densitometric analysis for COL-1 and IGF-1. Bars, mean ± SD. *P < 0.05. C, Quantitative reverse transcription-polymerase chain reaction was used for the determination of COL-1 mRNA expression and IGF-1 mRNA expression in the vaginal tissue of patients with POP and of the control group. D, The Pearson test revealed the relationship between the level of COL-1 mRNA and IGF-1 mRNA. E, Densitometric analysis for miR-19-3p expression in the vaginal tissue of patients with POP and the control group. F, Densitometric analysis for miR-19-3p expression in the degree III POP group and degree IV POP group. G, FISH staining was used to examine the expression of miR-19-3p. Nucleus was stained by DAPI. COL-1, collagen 1; IGF-1, insulin-like growth factor 1; mRNA, messenger RNA; POP, pelvic organ prolapse; POP-Q, pelvic organ prolapse quatitative.

The Western blot and qRT-PCR results showed that, compared with the control group, the expression of COL-1 and IGF-1 was decreased in the POP group (Figs. 1A–C). The Pearson test showed that there was a positive correlation between the COL-1 and IGF-1 expression. The miR-19-3p level was assayed by fluorescence in situ hybridization assay and qRT-PCR. The results showed that the miR-19-3p level was higher in the POP group (Figs. 1E, G). The expression of miR-19-3p was positively correlated with the severity of the prolapse (Fig. 1F).

### In the Cell Group With POP, the Expression of Akt/mTOR/p70S6K Pathway Protein Phosphorylation Was Decreased and Autophagy Was Increased

**FIGURE 2:**
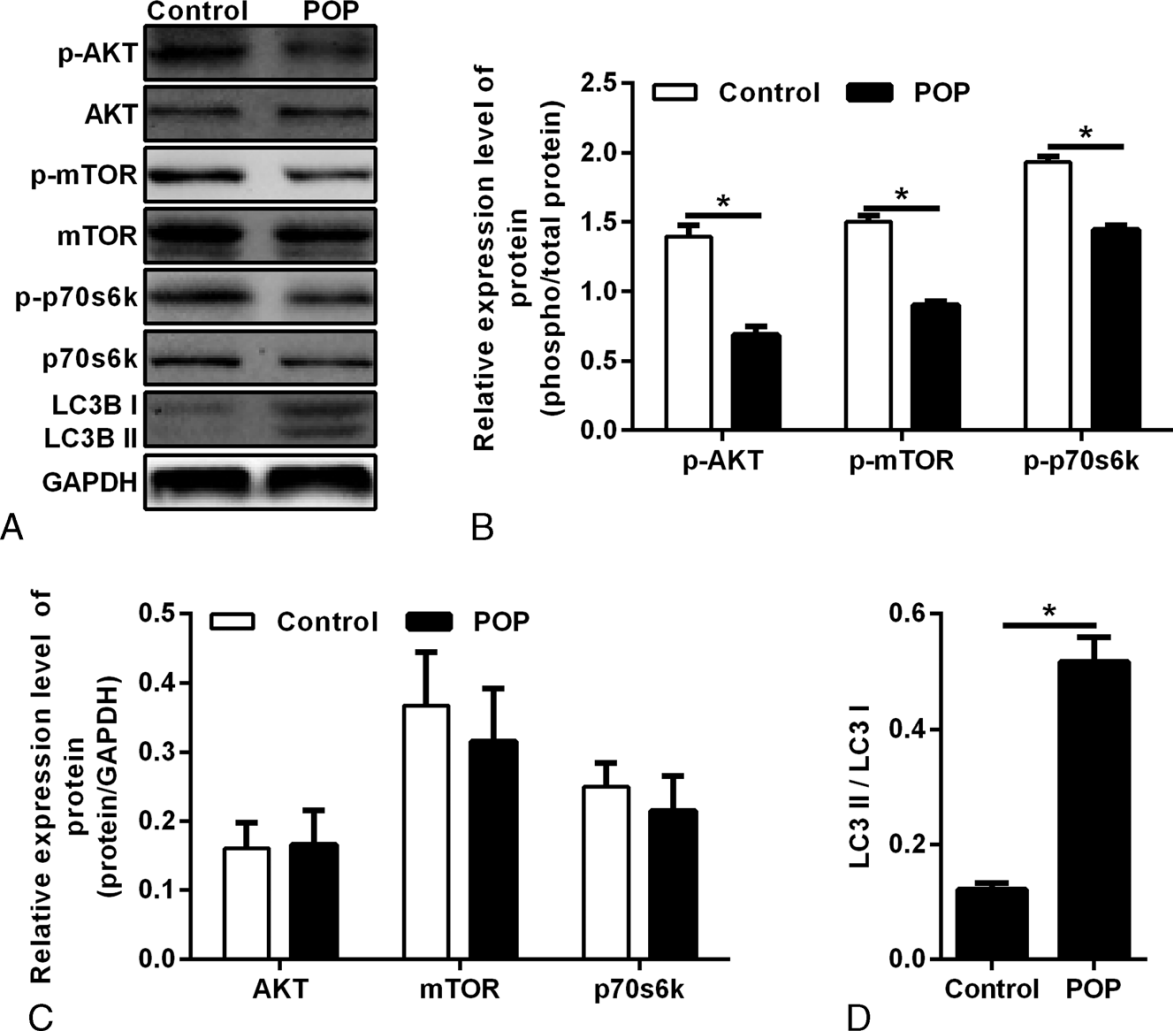
A, Protein expressions of AKT, p-AKT, mTOR, p-mTOR, p70S6K, and p-p70S6K were qualified by Western blotting in vaginal fibroblasts. B, Densitometric analysis for p-AKT, p-mTOR, and p-p70S6K. *P < 0.05. Bar, mean ± SD. C, The relative expression of AKT, mTOR, and p70S6K, which was normalized to GAPDH. D, Densitometric analysis for LC3II/LC3I, *P < 0.05. POP, pelvic organ prolapse.

To explore the potential mechanism of POP, we used the Western blot test to detect the expression of Akt, mTOR, p70S6K, and their phosphorylation indexes p-Akt, p-mTOR, and p-p70S6K in the fibroblasts of the prolapse group and control group. The results showed that the expression of p-Akt, p-mTOR, and p-p70S6K was decreased in the POP group (Figs. 2A, B), whereas the expression of Akt, mTOR, and p70S6K in the POP group showed no significant difference (Figs. 2A, C). The Western blot showed that the expression of LC3II/I was increased in the prolapse group, indicating a higher autophagy level.

### IGF-1 Promoted the Secretion of Type I Collagen in Vaginal Wall Fibroblasts, and an Increase in IGF-1 Inhibited the Autophagy Level of Cells

**FIGURE 3:**
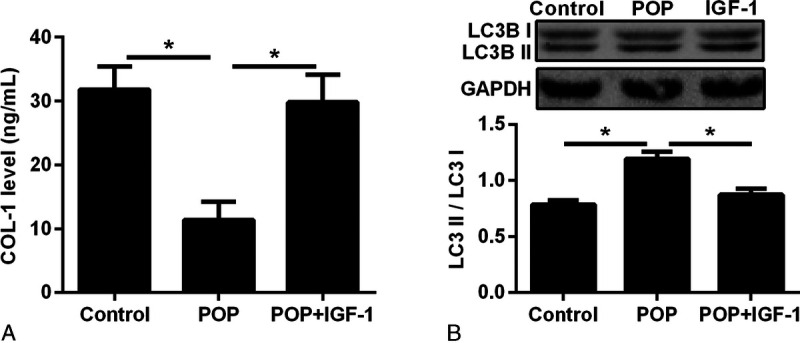
A, Enzyme-linked immunosorbent assay test for COL-1 in vaginal fibroblasts in the control group, POP group, and POP + IGF-1 group (IGF-1: 10 μg/L). *P < 0.05. B, Western blot and desitometric analysis for LC3B1 and LC3BII expression in vaginal fibroblasts in the control group, POP group, and POP + IGF-1 group (IGF-1: 10 μg/L). COL-1, collagen 1; IGF-1, insulin-like growth factor 1; POP, pelvic organ prolapse.

The ELISA experiment showed that compared with that in the control group, COL-1 secretion was decreased in the cells of the prolapse group. When IGF-1 was added to the cells of the prolapse group, the secretion of COL-1 protein was increased (Fig. 3A). The Western blot showed that the autophagy marker LC3II/I was increased in the POP group, indicating that the autophagy level was increased. After adding IGF-1 to the POP group, the expression of autophagy marker LC3II/I was decreased, indicating that the autophagy level was inhibited (Fig. 3B).

### IGF-1 Regulated COL-1 Secretion by Promoting the Akt/mTOR/p70S6K Pathway

**FIGURE 4:**
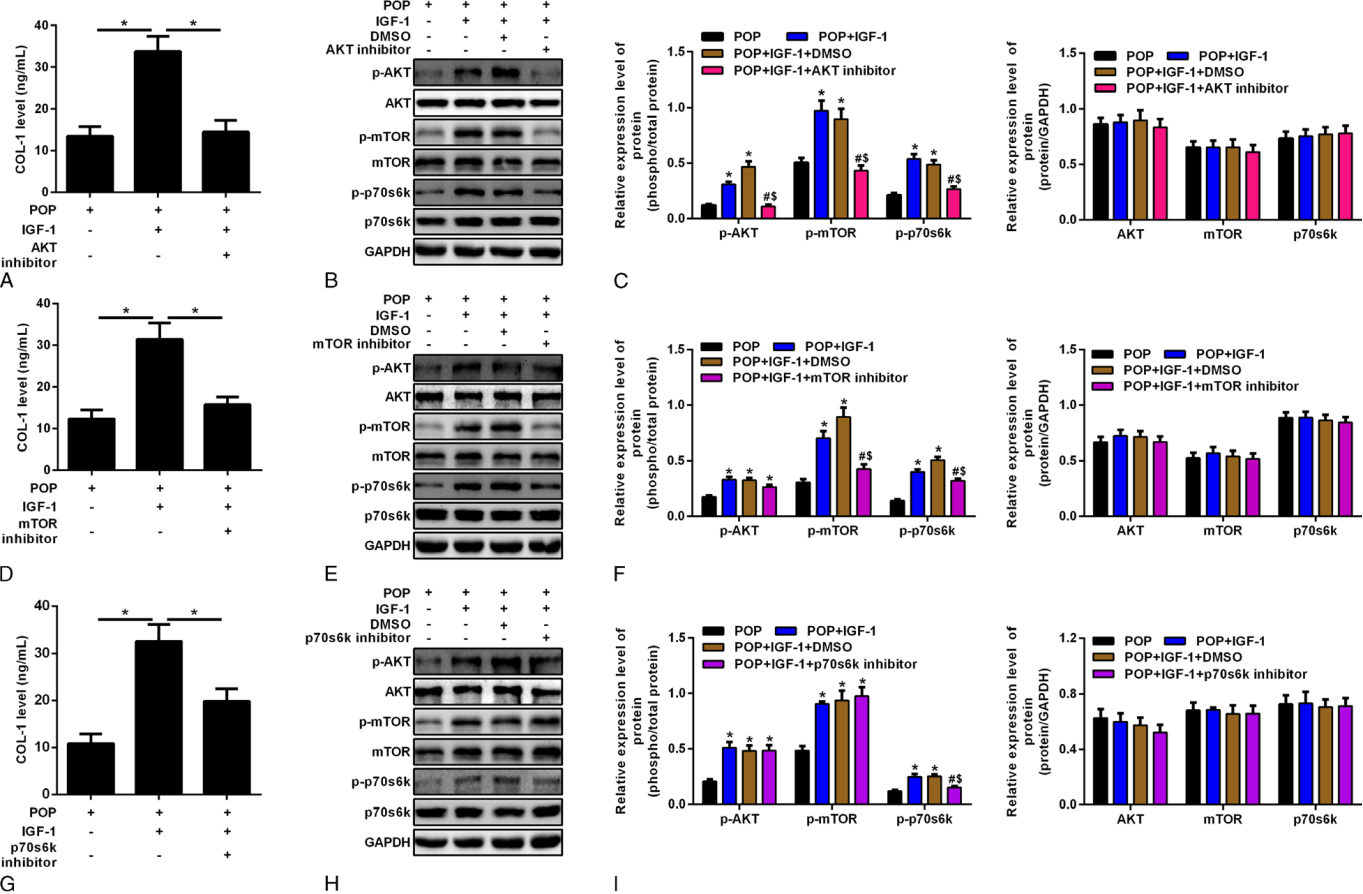
A, Vaginal fibroblasts, vaginal fibroblasts with IGF-1, and vaginal fibroblasts with IGF-1 and AKT inhibitor. Enzyme-linked immunosorbent assay for COL-1 level. *P < 0.05. B, Western blot for vaginal fibroblasts, with the addition of 50 μg/L IGF-1, DMSO, and AKT inhibitor (10 μM MK2206), respectively. C, Densitometric analysis for p-AKT, p-mTOR, p-p70S6K, AKT, mTOR, and p70S6K. *Means compared with the POP group, *P* < 0.05. ^#^Means compared with the POP + IGF-1 group, *P* < 0.05. ^s^Means compared with the POP+ IGF-1 + DMSO group, *P* < 0.05. D, Vaginal fibroblasts, vaginal fibroblasts with IGF-1, and vaginal fibroblasts with IGF-1 and mTOR inhibitor. Enzyme-linked immunosorbent assay for COL-1 level. *P < 0.05. E, Western blot for vaginal fibroblasts, with the addition with 10 μg/L IGF-1, DMSO, and mTOR inhibitor (10 μM rapamycin), respectively. F, Densitometric analysis for p-AKT, p-mTOR, and p-p70S6K, AKT, mTOR, and p70S6K. *Means compared with the POP group, *P* < 0.05. ^#^Means compared with the POP + IGF-1 group, *P* < 0.05. ^s^Means compared with the POP + IGF-1 + DMSO group, *P* < 0.05. G, Vaginal fibroblasts, vaginal fibroblasts with IGF-1, and vaginal fibroblasts with IGF-1 and p70S6K inhibitor. Enzyme-linked immunosorbent assay for COL-1 level. H, Western blot for vaginal fibroblasts, with the addition with 10 μg/L IGF-1, DMSO, and p70S6K inhibitor (10 μM LY2584702), respectively. I, Densitometric analysis for p-AKT, p-mTOR, and p-p70S6K, AKT, mTOR, and p70S6K. *Means compared with the POP group, *P* < 0.05. ^#^Means compared with the POP + IGF-1 group, *P* < 0.05. ^s^Means compared with the POP + IGF-1 + DMSO group, *P* < 0.05. COL-1, collagen 1; DMSO, dimethyl sulfoxide; IGF-1, insulin-like growth factor 1; mTOR, mammalian target of rapamycin; POP, pelvic organ prolapse.

After adding 50 μg/L IGF-1 to the fibroblasts of the prolapse group, ELISA detected the increase in COL-1 secretion. After adding the AKT inhibitor (10 μM MK2206), mTOR inhibitor (10 μM rapamycin), and p70S6K inhibitor (10 μM LY2584702) separately, it was observed that the expression of COL-1 was decreased compared with that without the corresponding inhibitors (Figs. 4A, D, G). The Western blot showed that the expression of p-Akt, p-mTOR, and p-p70S6K in the downstream pathway was increased after adding 50 μg/L IGF-1. The expression of p-Akt, p-mTOR, and p-p70S6K was decreased significantly when the Akt inhibitor was added, but there was no significant difference in the expression of Akt, mTOR, and p70S6K (Figs. 4B, C). After adding rapamycin, the expression of p-mTOR and p-p70S6K was decreased significantly, but no significant difference was found in the other indexes (Figs. 4E, F). After adding the p70S6K inhibitor, the expression of p-p70S6K was decreased significantly, and there was no significant difference in the other indexes (Figs. 4H, I).

### miR-19-3p Increase Promoted Autophagy

**FIGURE 5:**
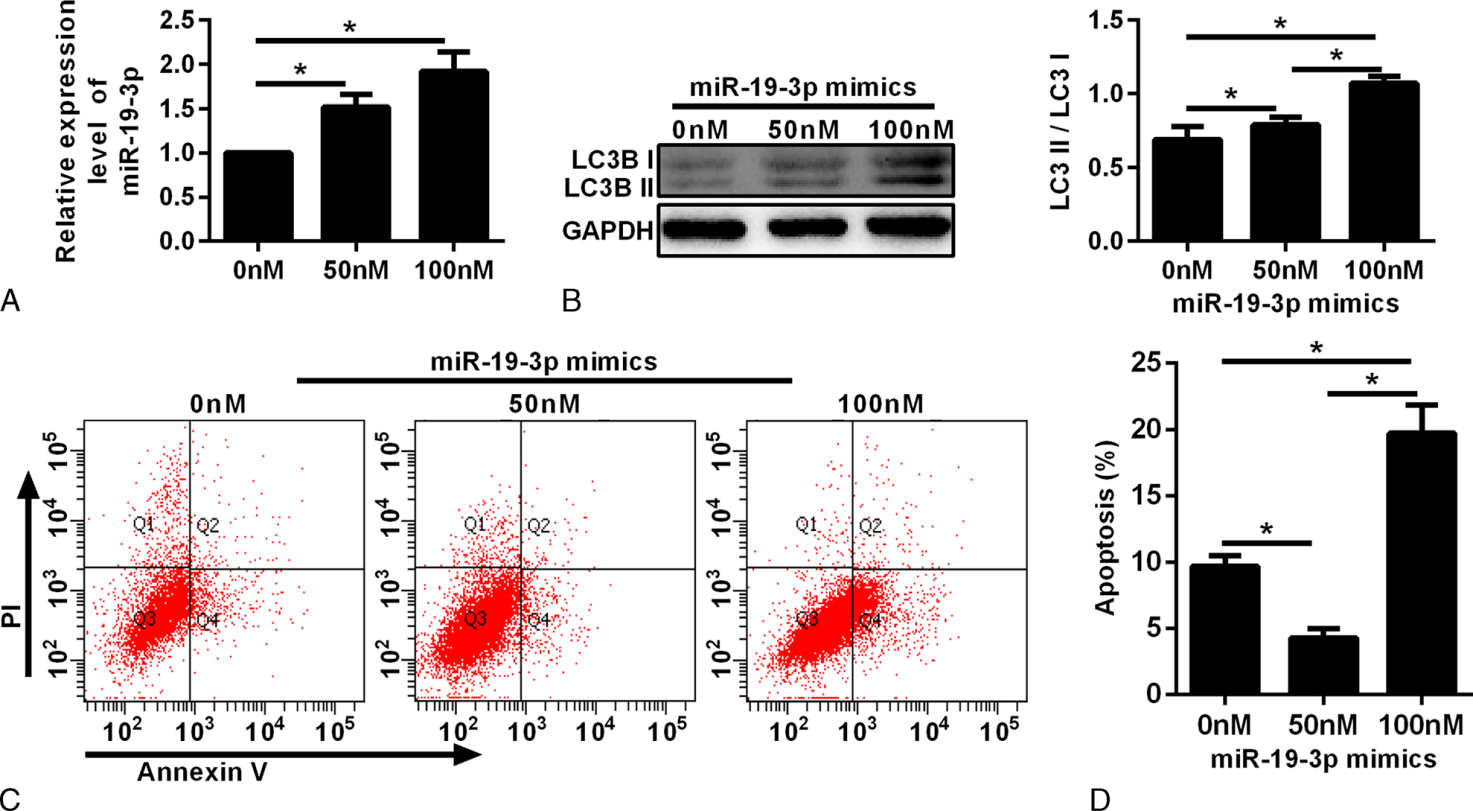
The effect of miR-19-3p on the apoptosis rate and autophagy level of vaginal fibroblast cells. A, Quantitative reverse transcription-polymerase chain reactions were conducted to detect miR-19-3p messenger RNA expression under different concentrations of miR-19-3p mimic transfection. B, Western blot and densitometric analysis for LC3I and LC3II with 0 nM, 50 nM, and 100 nM miR-19-3p mimic transfection. C, Annexin V–FITC/PI double-stained cells were examined by flow cytometry. D, Analysis of the apoptotic rate. *P < 0.05.

The expression of miR-19-3p messenger RNA was increased with an increase in the transfection concentration of miR-19-3p mimics (Fig. 5A). The autophagy level was observed after different concentrations of miR-19-3p mimics were transfected. With the increase in miR-19-3p mimic transfection concentration, LC3II/I was increased, indicating an increase in the autophagy level (Figs. 5B, C). Apoptosis was detected by flow cytometry. With an increasing concentration of miR-19-3p mimics, the level of apoptosis was decreased first and then it increased (Figs. 5D, E).

### miR-19-3p Negatively Regulated the Akt/mTOR/p70S6K Pathway and Inhibited COL-1 Secretion

**FIGURE 6:**
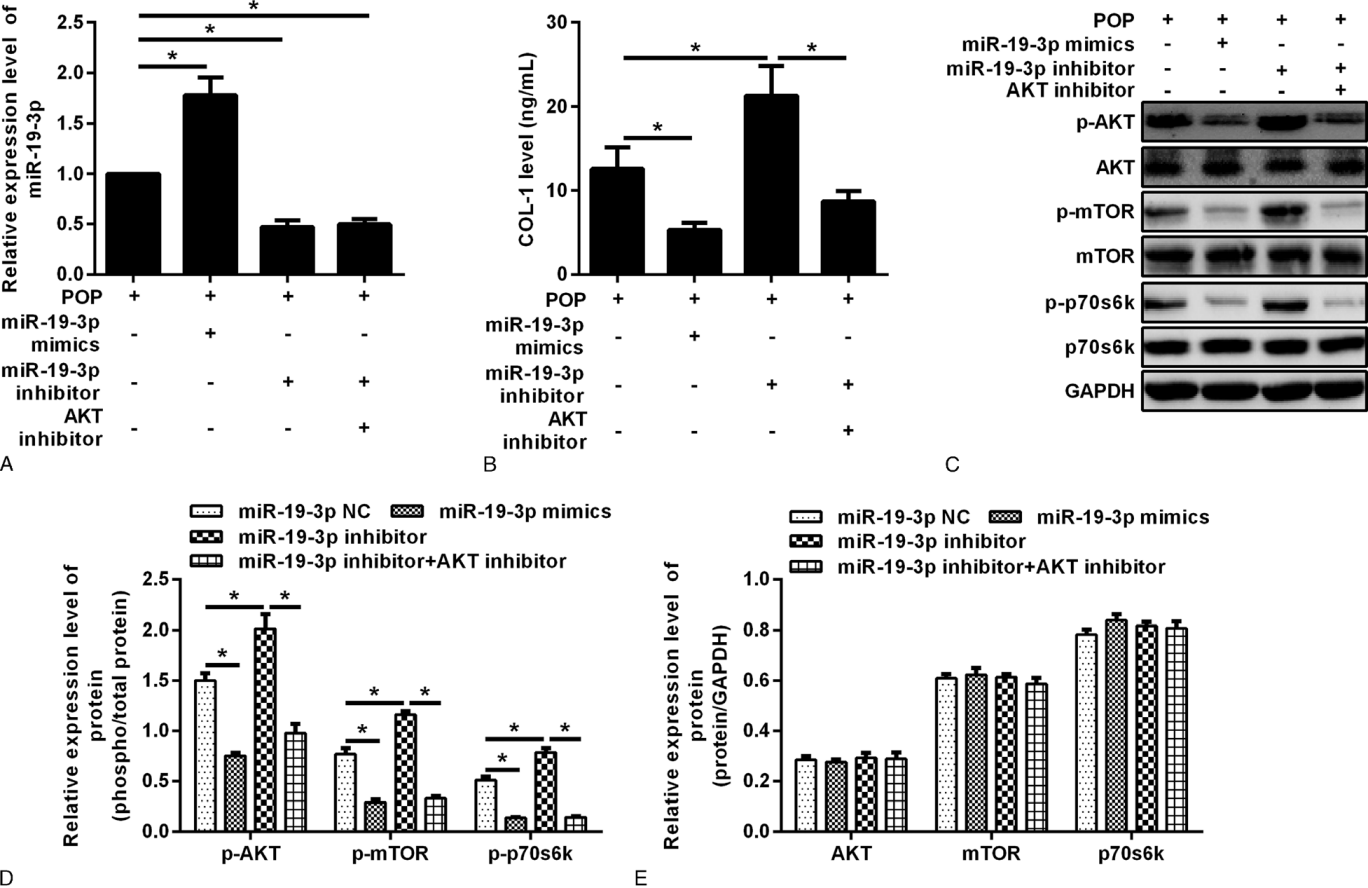
Fibroblasts were transfected with miR-19-3p NC, miR-19-3p mimic, miR-19-3p inhibitor, and miR-19-3p inhibitor + AKT inhibitor, respectively. A, The expression of miR-19-3p was detected by quantitative reverse transcription-polymerase chain reaction. *P < 0.05. B, Col-1 expression level was determined by enzyme-linked immunosorbent assay. *P* < 0.05. C, Western blot for AKT, mTOR, p70S6K, p-AKT, p-mTOR, and p-p70S6K in the 4 groups. D, Densitometric analysis for p-AKT, p-mTOR, and p-p70S6K in these groups. *P < 0.05. E, Densitometric analysis for AKT, mTOR, and p70S6K in these groups. * *P* < 0.05. mTOR, mammalian target of rapamycin; NC, negative control.

Vaginal fibroblasts from the POP group were transfected with miR-19-3p mimics, the miR-19-3p inhibitor, and the miR-19-3p inhibitor with the AKT inhibitor. The expression of miR-19-3p was detected by qRT-PCR. As shown in Figure 6A, the expression of miR-19-3p was increased after the transfer of miR-19-3p mimics, and it was decreased after the transfer of the miR-19-3p inhibitor. After the simultaneous transfection of the miR-19-3p inhibitor and Akt inhibitor simultaneously, the expression of miR-19-3p was decreased compared with that in the control group, but there was no significant difference compared with that of pure transfer in the miR-19-3p inhibitor group.

We observed the expression of COL-1 by ELISA. The expression of COL-1 was decreased after the miR-19-3p mimics were transferred, and it was increased after the miR-19-3p inhibitor was transferred. The expression of COL-1 after the miR-19-3p inhibitor transfected with Akt inhibitor was significantly lower than that after the miR-19-3p inhibitor transfected alone (Fig. 6B).

We observed the expression of the Akt mTOR p70S6K pathway protein after different treatments by Western blot. The results showed that the expression of p-Akt, p-mTOR, and p-p70S6K was significantly decreased after the miR-19-3p mimics were transferred, but there was no significant difference in Akt, mTOR, and P70S6K expression. After transfection of the miR-19-3p inhibitor, the expression of p-Akt, p-mTOR, and p-p70S6K was increased significantly, but there was no significant difference in the expression of Akt, mTOR, and p70S6K. Compared with the miR-19-3p inhibitor alone, p-Akt, p-mTOR, and p-p70S6K were significantly reduced after transfection of the miR-19-3p inhibitor and Akt inhibitor simultaneously (Figs. 6C–E).

### miR-19-3p Regulated IGF-1 Expression by Direct Target Binding

**FIGURE 7:**
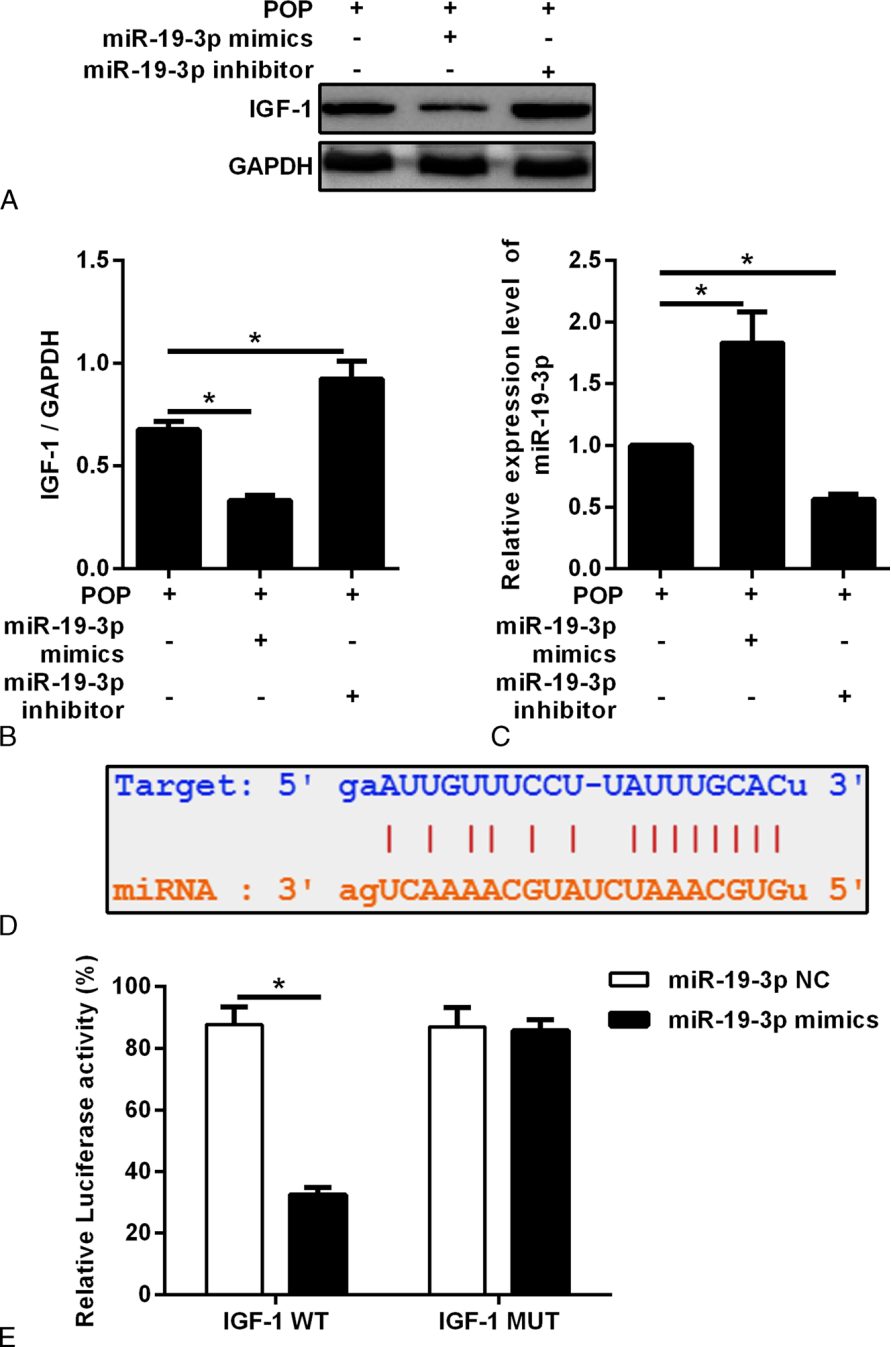
A, Vaginal fibroblasts were transfected with the miR-19-3p mimic and miR-19-3p inhibitor. The protein expression of IGF-1 was detected. B, Densitometric analysis of IGF-1. Results were normalized with GAPDH. *P < 0.05. C, Densitometric analysis of miR-19-3p. Results were normalized with GAPDH. *P < 0.05. D, The binding site of miR-19-3p to the 3′-untranslated region of IGF-1. E, The relative luciferase activities of IGF-1 WT and IGF-1 MUT were detected in the miR-19-3p NC and miR-19-3p mimic group using the luciferase reporter gene assay. IGF-1, insulin-like growth factor 1; NC, negative control for miR-19-3p; MUT, mutant type; WT, wild type; POP, pelvic organ prolapse.

IGF-1 expression was detected in the miR-19-3p mimic group, miR-19-3p inhibitor group, and miR-19-3p NC group. The results showed that the expression of miR-19-3p was decreased and that of IGF-1 was increased in the miR-19-3p inhibitor group compared with the miR-19-3p NC group. In the miR-19-3p mimic group, the expression of miR-19-3p was increased and that of IGF-1 was decreased (Figs. 7A–C).

To further clarify the interaction between miR-19-3p and IGF-1, we conducted luciferase experiments. The results showed that luciferase activity was significantly decreased in the cells cotransfected with the miR-19-3p mimic and IGF1-WT compared with the cells cotransfected with miR-19-3p NC and the IGF1-WT group. There were no significant changes in luciferase activity in the cells cotransfected with the IGF1-MUT vector. This result further proves that miR-19-3p is targeted to the 3′-UTR of IGF-1 (Figs. 7D, E).

## DISCUSSION

In our study, we found that there was a positive correlation between increased degree of prolapse and increased expression of miR-19-3p. miRNA dysregulation plays an important role in many pathological processes such as cell proliferation and differentiation. miR-19-3p has been found to be associated with fibrosis in Crohn disease. MiRNAs can be used as diagnostic markers or therapeutic targets in some specific condition. Pelvic organ prolapse is a condition associated with a decrease in collagen 1 secretion. A characteristic of this kind of condition is that it can delay the development of the disease through pelvic floor function exercises in the early stages. However, if the condition progresses to become more serious, it may need to be solved by surgery. Therefore, looking for potential targets for early diagnosis to prevent POP is important.

In our study, we found that, after an overexpression of miR-19-3p, the expression of p-Akt, p-mTOR, and p-p70S6K was decreased, autophagy was increased, and COL-1 expression was decreased. After inhibiting the expression of miR-19-3p, the expression of p-Akt, p-mTOR, and p-p70S6K was increased, autophagy was decreased, and COL-1 expression was increased. At the same time, in this study, we found that IGF-1 can activate the Akt/mTOR/p70S6K pathway, inhibit the level of autophagy, and promote an increase in COL-1 expression. Luciferase experiments revealed that IGF-1 was the direct target of miR-19-3p. All of these results support the claim that miR-19-3p promotes vaginal fibroblast autophagy and apoptosis in POP through the Akt/mTOR/p70S6K pathway by targeting IGF-1.

In histological experiments, we found that the expression of COL-1 was significantly decreased in the vaginal wall tissue of patients with POP, which is consistent with the results of Gede et al.^16^ It has been suggested that POP may be related to weakness of pelvic floor ligaments and a decrease in COL-1 expression. In addition, we found that the content of IGF-1 was decreased in the vaginal wall of patients with POP, and the expression level of IGF-1 was positively correlated with the expression level of COL-1. It has been suggested that IGF-1 plays an important role in promoting the collagen secretion of fibroblasts. Previous studies have shown that IGF-1 messenger RNA expression is increased in fibrotic tissues.^17^ Provenzano et al^18^ have shown that IGF-1 can help the recovery of ligament tissue damage by promoting the secretion of type I collagen and type III collagen. Prokop et al^19^ found that IGF-1 can promote the secretion of collagen in fibroblasts. The inhibition of IGF receptor expression can inhibit the synthesis of COL-1, thus inhibiting tissue fibrosis. A clinical study on urinary incontinence by Ozbek et al^20^ showed that the expression of IGF-1 was decreased in the serum of patients with urinary incontinence. Another basic study by Sumino et al^21^ showed that the expression of IGF-1 was decreased in the transverse striated sphincter of the urinary tract in mice with incontinence. These results suggest that decreased expression of IGF-1 may have an effect on the supporting function of ligaments of the pelvic floor. Up to now, there has been no exploration of IGF-1 and POP. In this study, we found that the expression of IGF-1 decreased in the vaginal wall of patients with POP. An increase in the IGF-1 content in fibroblasts can promote COL-1 secretion. These results may suggest that IGF-1 plays an important role in the pathogenesis of pelvic floor dysfunction.

The Akt/mTOR/p70S6K pathway plays an important role in the proliferation, growth, and differentiation of fibroblasts. mTOR is a highly conserved protein family and a key protein in cell proliferation, growth, and differentiation. It has been found that the mTOR pathway can improve cell viability by inhibiting excessive autophagy.^22^ In this study, we found that, compared with the control group, the expression of p-Akt, p-mTOR, and p-p70S6K was decreased in the vaginal wall fibroblasts extracted from the prolapse group, the expression of LC3II/I, an autophagy marker, was significantly increased, and the expression of COL-1 was decreased. Also, this effect could be inhibited by Akt and mTOR inhibitors. After adding the Akt inhibitor MK2206, the expression level of COL-1 decreased and the downstream pathway protein phosphorylation level decreased. When rapamycin, an inhibitor of mTOR, was added, the expression of COL-1 decreased and the phosphorylation level of the downstream pathway decreased. After adding LY2584702, a p70S6K inhibitor, the expression of COL-1 decreased. Therefore, the expression of COL-1 is regulated by the Akt/mTOR/p70S6K pathway.

In other studies, the miR-19 family is believed to be involved in the regulation of fibrosis.^23^ We observed the effect of miR-19-3p on the expression of COL-1 in fibroblasts. This is consistent with the findings of the study by Lewis et al,^12^ which suggests that the expression of miR-19-3p in patients with Crohn disease with severe intestinal fibrosis is significantly reduced. In this study, we found that the level of autophagy was increased, the level of apoptosis was first decreased and then increased, and the expression of COL-1 was decreased after transfection into fibroblasts with miR-19-3p mimics. All of these findings suggested that miR-19-3p might affect the expression of COL-1 gene. Moreover, upregulation of miR-19-3p can inhibit the expression of p-Akt, p-mTOR, and p-p70S6K, whereas downregulation of miR-19-3p can promote the expression of p-Akt, p-mTOR, and p-p70S6K. These results suggest that miR-19-3p may affect the autophagy level of fibroblasts through the Akt/mTOR/p70S6K pathway, thus affecting the secretion of COL-1 in fibroblasts.

To further determine the effect of miR-19-3p on COL-1 secretion by vaginal fibroblasts, it is very important to identify the target gene of miR-19-3p. We found that compared with the miR-19-3p NC group, IGF-1 expression was increased in the miR-19-3p inhibitor group, and it was decreased in the miR-19-3p mimic group. We used the luciferase method to study the relationship between miR-19-3p and IGF-1. The results showed that miR-19-3p played a role by regulating the 3′-UTR region of IGF-1 receptor. Our study found that miR-19-3p controlled the intracellular signal transduction pathway and it has an important effect on the secretion of COL-1 by vaginal wall fibroblasts. These results show that miR-19-3p can promote vaginal fibroblast autophagy and apoptosis in POP through the AKT/mTOR/p70S6K pathway via target IGF-1.

miR-19-3p may be a potential target for early diagnosis and prevention for POP. The whole experimental design uses clinical specimens for research, which is closer to clinical application than are animal models, but the results should be verified by further clinical studies.
